# Hippocampal profiling: Localized magnetic resonance imaging volumetry and T2 relaxometry for hippocampal sclerosis

**DOI:** 10.1111/epi.16416

**Published:** 2019-12-24

**Authors:** Sjoerd B. Vos, Gavin P. Winston, Olivia Goodkin, Hugh G. Pemberton, Frederik Barkhof, Ferran Prados, Marian Galovic, Matthias Koepp, Sebastien Ourselin, M. Jorge Cardoso, John S. Duncan

**Affiliations:** ^1^ Centre for Medical Image Computing University College London London UK; ^2^ Epilepsy Society MRI Unit Chalfont St Peter UK; ^3^ Department of Clinical and Experimental Epilepsy University College London London UK; ^4^ Neuroradiological Academic Unit UCL Queen Square Institute of Neurology University College London London UK; ^5^ Division of Neurology Department of Medicine Queen’s University Kingston Canada; ^6^ Dementia Research Centre UCL Queen Square Institute of Neurology University College London London UK; ^7^ Lysholm Department of Neuroradiology National Hospital for Neurology and Neurosurgery National Health Service Foundation Trust London UK; ^8^ Queen Square Multiple Sclerosis Centre Department of Neuroinflammation Faculty of Brain Sciences UCL Queen Square Institute of Neurology University College London London UK; ^9^ Department of Radiology and Nuclear Medicine VU University Medical Center Amsterdam the Netherlands; ^10^ eHealth Center Open University of Catalonia Barcelona Spain; ^11^ Department of Neurology University Hospital Zurich Zurich Switzerland; ^12^ School of Biomedical Engineering and Imaging Sciences King’s College London London UK

**Keywords:** hippocampal sclerosis, hippocampal volumetry, neuroimaging, relaxometry

## Abstract

**Objective:**

Hippocampal sclerosis (HS) is the most common cause of drug‐resistant temporal lobe epilepsy, and its accurate detection is important to guide epilepsy surgery. Radiological features of HS include hippocampal volume loss and increased T2 signal, which can both be quantified to help improve detection. In this work, we extend these quantitative methods to generate cross‐sectional area and T2 profiles along the hippocampal long axis to improve the localization of hippocampal abnormalities.

**Methods:**

T1‐weighted and T2 relaxometry data from 69 HS patients (32 left, 32 right, 5 bilateral) and 111 healthy controls were acquired on a 3‐T magnetic resonance imaging (MRI) scanner. Automated hippocampal segmentation and T2 relaxometry were performed and used to calculate whole‐hippocampal volumes and to estimate quantitative T2 (qT2) values. By generating a group template from the controls, and aligning this so that the hippocampal long axes were along the anterior‐posterior axis, we were able to calculate hippocampal cross‐sectional area and qT2 by a slicewise method to localize any volume loss or T2 hyperintensity. Individual patient profiles were compared with normative data generated from the healthy controls.

**Results:**

Profiling of hippocampal volumetric and qT2 data could be performed automatically and reproducibly. HS patients commonly showed widespread decreases in volume and increases in T2 along the length of the affected hippocampus, and focal changes may also be identified. Patterns of atrophy and T2 increase in the left hippocampus were similar between left, right, and bilateral HS. These profiles have potential to distinguish between sclerosis affecting volume and qT2 in the whole or parts of the hippocampus, and may aid the radiological diagnosis in uncertain cases or cases with subtle or focal abnormalities where standard whole‐hippocampal measurements yield normal values.

**Significance:**

Hippocampal profiling of volumetry and qT2 values can help spatially localize hippocampal MRI abnormalities and work toward improved sensitivity of subtle focal lesions.

**Key Points**

- HS is radiologically characterized by atrophy and increased T2‐weighted signal
- Quantification of these features improves sensitivity, but utility may be limited if only one value is given for the whole hippocampus
- Localized quantification through profiling along the anterior‐posterior hippocampal axis can be automated reliably and reproducibly
- Hippocampal profiling can be used as a tool to improve characterization and possibly diagnosis of HS, especially in subtle or focal HS

## 1 INTRODUCTION

Hippocampal sclerosis (HS) is the most common cause of medically refractory temporal lobe epilepsy (TLE),^1^ and surgical resection has a high chance of achieving seizure remission.^2^, ^3^ HS typically manifests radiologically as loss of volume, loss of internal architecture, and T2 hyperintensity.^4^ Magnetic resonance imaging (MRI) protocols used to visualize HS include high‐resolution T1‐weighted imaging to detect volume loss (atrophy) and T2‐weighted or T2–fluid‐attenuated inversion recovery (FLAIR) scans showing increased T2 signal.^5^ Atrophy has been shown to correlate with laterality of seizure onset^6^ and seizure outcome after anterior temporal lobe resection.^7^ T2 abnormalities can be present even if there is no hippocampal volume reduction, and have been reported as the most consistent MRI finding in HS.^8^

Quantitative evaluation of atrophy and T2 hyperintensity, using hippocampal volumetry and T2 relaxometry, can yield a higher sensitivity to detect HS than qualitative visual inspection.^5^, ^9^, ^10^ Volumetry can help distinguish between normal and abnormal volumes in cases that may be difficult to classify visually.^11^ T2 relaxometry, also called quantitative T2 (qT2) measurements, can be used to give an objective reflection of the T2 relaxation properties of the tissue.

In recent years, both volumetry^12^ and T2 relaxometry^13^ methods have been automated, enabling routine clinical use in comparing a patient's individual values to a normative database of healthy control subjects. These methods, however, only yield a single volume and qT2 value per hippocampus and may be insensitive to subtle focal abnormalities.^14^ In this work, we present an automated method to generate subject‐specific localized volume and qT2 profiles along the hippocampal long axis to overcome this limitation. The software is made freely available as a modification of the online web‐based HIPPOSEG service (http://niftyweb.cs.ucl.ac.uk/program.php?p=HIPPOPROF).^12^, ^15^

## 2 MATERIALS AND METHODS

### 2.1 Data acquisition

Subjects underwent imaging on a 3‐T GE Discovery MR750 scanner with a 32‐channel coil. Sequences included a three‐dimensional (3D) T1‐weighted inversion‐recovery fast spoiled gradient recalled echo (echo time [TE] 3.1 milliseconds, repetition time [TR] = 7.4 milliseconds, inversion time = 400 milliseconds, field of view [FOV] = 224 × 256 × 256 mm, matrix = 224 × 256 × 256, voxel size = 1.00 × 1.00 × 1.00 mm = 1.00 mm^3^, parallel imaging acceleration factor = 2) and a coronal dual‐echo fast recovery fast spin echo proton‐density/T2‐weighted sequence (TE = 30/119 milliseconds, TR = 7600 milliseconds, FOV = 220 × 220 mm, matrix = 512 × 512, slice thickness = 4 mm, voxel size = 0.43 × 0.43 × 4.00 mm = 0.74 mm^3^, SENSE factor = 2).

For patients, this was part of their routine clinical MRI protocol, which also included a 3D T2‐FLAIR^16^ that was presented to radiologists for reporting.

### 2.2 Subjects

We expanded our healthy control group with respect to Winston et al^13^ to 111 healthy controls (age mean [μ] and standard deviation [σ] = 40.0 ± 12.8, range = 17.0‐66.6 years; 52 male [M]/59 female [F]) without any history of neurologic or psychiatric disease, from previously scanned subjects. All controls were scanned on the same scanner within a 30‐month time frame in 2015‐2018. Twenty controls were rescanned within 1 year of their first scan to evaluate reproducibility. The study was considered a service improvement using clinically acquired data by the National Hospital for Neurology and Neurosurgery and the Institute of Neurology Joint Research Ethics Committee. Informed written consent was obtained from control subjects.

We included 69 patients (age μ ± σ = 42.7 ± 14.7, range = 18.0‐76 years; 31 M/38 F) who had undergone brain MRI with an epilepsy protocol as part of routine clinical practice for TLE at the Epilepsy Society MRI Unit, Chalfont St Peter, between 2015 and 2018, and who had been reported by a neuroradiologist as showing unilateral HS or bilateral HS (BHS) on visual assessment and concordant with neurological examination. This consisted of 32 left HS (LHS), 32 right HS (RHS), and five BHS.

Furthermore, we reviewed all cases having undergone TLE surgery at our center who had pathologically confirmed HS but without mention of HS in the radiological report. Five patients had had all the MRI sequences mentioned above (see Table S1 for details).

### 2.3 Image processing

Automated volumetry and T2 relaxometry were performed as described previously (Winston et al, 2013 and 2017, respectively).^12,13^ In brief, this used a multi‐atlas–based algorithm for the segmentation (STEPS)^17^ using the 3D T1 images, which was then coregistered to the proton density (PD)/T2 scan and used as a mask to obtain qT2 values within only the hippocampus. Hippocampal volumes were corrected for total intracranial volume (TIV) as in Winston et al.^13^

The processing to obtain profiles along the anterior‐posterior (AP) axis of the hippocampus then consists of (1) the generation of a group template, (2) registration of individual scans, (3) creating a normative database, (4) creating the disease group average, and (5) comparing individual subject scans to that normative database.

#### 2.3.1 Group template generation

**Figure 1:**
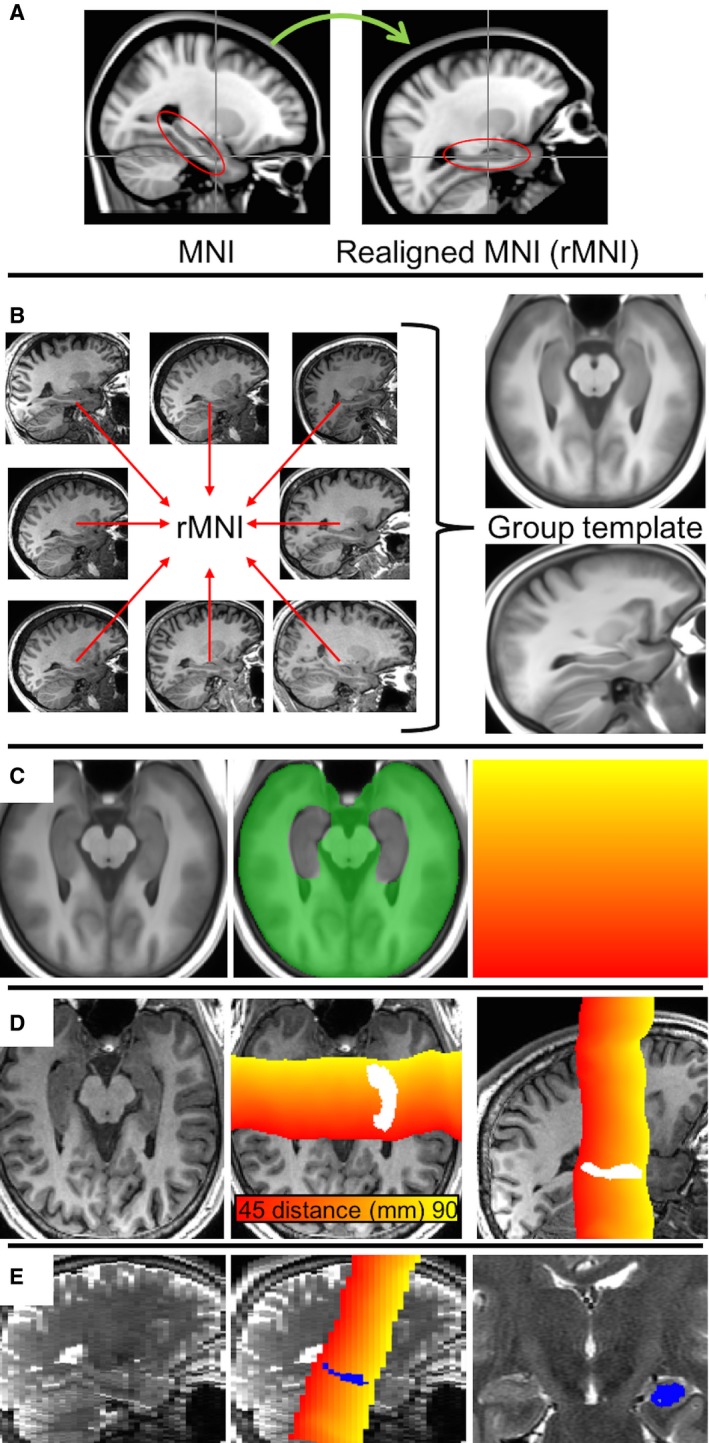
A, The Montreal Neurological Institute (MNI)‐152 template is reoriented so that the hippocampi are along the anterior‐posterior (AP) axis (realigned MRI). B, All healthy controls were registered to this in a groupwise manner to generate a group template image. C, D, A brain mask (green) excluding the hippocampi is generated for the group template to drive the nonlinear registration between template (C) and subject (D). The A‐P distance map, defined on the template (C, right) is resampled to the subject space (D), shown only in the range around the hippocampus to emphasize the distance differences, including segmentation shown axially (middle) and sagittally (right). At each coronal slice of the segmentation, the distance map values are averaged to generate an average A‐P distance per slice. E, Sagittal view of the T2 map (left), including distance map and segmentation (middle) and coronal view including segmentation (right)

The hippocampal regions of interest (ROIs) from the Harvard‐Oxford atlas of the Montreal Neurological Institute (MNI)‐152 template were extracted, and using principal component analysis, their long axis was obtained. The MNI‐152 template was then reoriented so that the hippocampal ROIs were along the AP axis (Figure 1A). The 3D T1 scans of all 111 healthy controls were then registered to this rotated MNI template in an iterative manner using 10 affine registration steps and 10 nonlinear (fast free‐form deformation) registration steps to obtain a population‐specific group average template. All registrations were done with the open source NiftyReg software package (Figure 1B).^18^, ^19^ For use in the later steps, a distance map along the AP axis of this template was generated (Figure 1C, right), from the most posterior slice to the most anterior slice (distances = 1‐218 mm, respectively). The hippocampal segmentations from the healthy controls were transformed to the group template with the obtained transformation parameters. Voxels were included in the groupwise hippocampal masks when they were included in at least half the individual segmentations.

#### 2.3.2 Registration of individual scan to template

Each subject's 3D T1 scan was registered to the group template by a rigid registration to ensure similar orientations across subjects for visual comparison and symmetric alignment of the hippocampal long axes with the AP axis. For accurate matching to the group template, each subject's 3D T1 was nonrigidly registered to the group template by first a full affine registration to account for scaling between different head sizes, followed by a nonlinear registration to account for morphological differences between subjects. Importantly, this nonlinear registration was optimized by only evaluating the cost‐function in the registration in the brain excluding the hippocampal segmentations from the template; this was done to avoid influence of atrophy or other pathology on the registration (Figure 1C, middle). The registrations were then inverted to obtain the transformation from the template to the subject's T1 scan. The distance map from step 1 was then transformed to the subject's T1. For each coronal slice in the subject's hippocampal segmentation, the distances were averaged to obtain a mapping from the AP location in the template to the subject's scan (Figure 1D). Cross‐sectional areas (CSAs)—corrected for TIV—were calculated for each slice, which together with the distances provides a single‐subject profile.

For the T2 relaxometry, the transformation from the template to the T1 was concatenated with the rigid transformation from the T1 to the PD/T2 scan.^13^ Similarly, the template distance map was transformed to obtain a mapping of location and averaged within the hippocampal segmentation in the PD/T2 space (Figure 1E).

#### 2.3.3 Normative database generation

To generate a normative range with which to compare individual patient profiles, all 111 healthy volunteers’ CSA and T2 profiles were used. At steps of 1 mm along the AP axis, μ and σ of the hippocampal CSA and qT2 were calculated using a kernel density estimator. These were used to generate a normative range (μ ± 1.96 σ) of CSA and qT2 at each point along the AP axis of the hippocampus.

#### 2.3.4 HS group averages

To compare TLE patient groups to controls in a groupwise fashion, step 3 was repeated for each of the LHS, RHS, and BHS groups.

#### 2.3.5 Comparison of individual profiles

To compare individual subject profiles to the normative database from step 3, the same registrations as in step 2 were used. For the CSA, which originates from a 1‐mm isotropic scan, the subject's profile is shown as a continuous profile to be compared to the normative data (μ ± 1.96 σ). For the qT2 values, calculated from a 2D acquisition with 4‐mm slice thickness, the data points from each slice are shown.

### 2.4 Interscan reproducibility

To investigate scan‐rescan reproducibility of the profiles, 20 controls that were scanned twice were processed and compared. The CSA and T2 profiles were subtracted from each other at each point, and the same kernel density estimation method as above was used to estimate the scan‐rescan variability by estimating differences between CSA and T2 values from the two scans at each point along the AP axis.

### 2.5 Distinction of regions

We further divided the hippocampus into three distinct regions—its head, body, and tail—to quantify the volumes and qT2 values regionally. We defined the body section as that which has a stable CSA in our normative data, where we define stable as a change in CSA < 2 mm^2^/mm. This results in head, body, and tail sections of 24/15/8 mm in length for the left and 26/12/8 mm for the right hippocampus. Volumes of these regions were calculated by summing the CSA from each section. qT2 values per region were calculated by averaging the slicewise qT2 values per region weighted by the volume of the segmentation in each slice.

### 2.6 Statistical interpretation

Whole‐hippocampal volumes and qT2s were compared using analysis of variance between the four groups, and Tukey honest significant difference test was used to explore which groups are significantly different if group‐level differences were detected.

Statistical comparisons were performed at each point along the AP axis comparing the normative range (from the 111 control subjects) to the three patient groups. For group comparisons, all CSA and qT2 profiles were linearly interpolated to compare at every millimeter along the AP axis. Using two‐sample *t* tests, each point along the profile was compared between all control profiles and patients from each group, using *P* < .01 with Bonferroni correction for the number of points along the profile. For evaluating differences in the tail, body, and head, this was done at *P* < .01/3. For individual comparisons, any point along the AP axis was deemed atrophic if the subject profile fell below the normal range (ie, CSA < μ − 1.96 σ). For the T2 measurements, this was if the subject profile showed higher qT2 than the normal range (ie, qT2 > μ + 1.96 σ). The percentages of individuals with an abnormal profile were calculated for each point along the normative profile.

## 3 RESULTS

**Figure 2:**
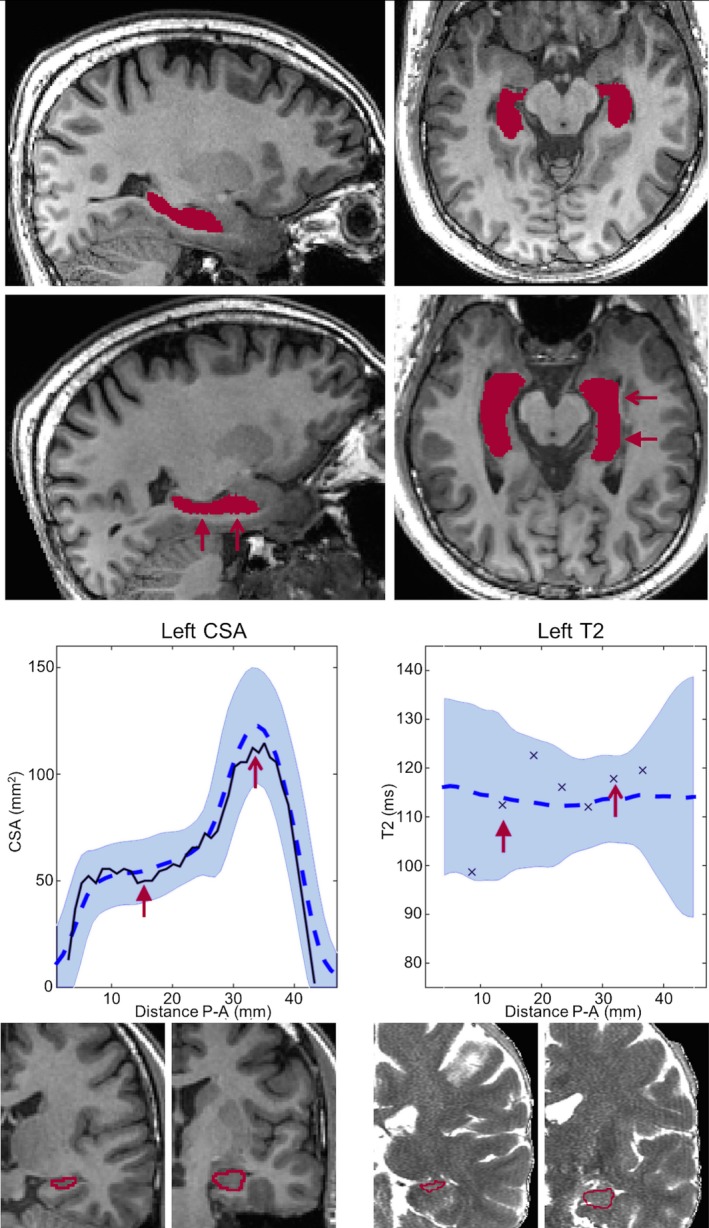
A raw three‐dimensional T1 image of a healthy control with hippocampal segmentations is shown in the top row, in a sagittal (left panel) and axial (right panel) view. After rigid rotation to the group template (second row), the hippocampal long axis is along the posterior‐anterior (P‐A) axis. The cross‐sectional area (CSA) and T2 profiles are shown in the third row with a black line and black crosses, respectively, over the normative range (blue shaded area). The red arrows indicate cuts through the hippocampus at the body and the head, as shown in the bottom row (T1 on left, T2 map on right)

An example profile of a healthy control is shown in Figure 2, which shows two example cross‐sectional cuts through the hippocampal segmentation at the head and body. The closest corresponding slices from the T2 map are shown as well.

**Figure 3:**
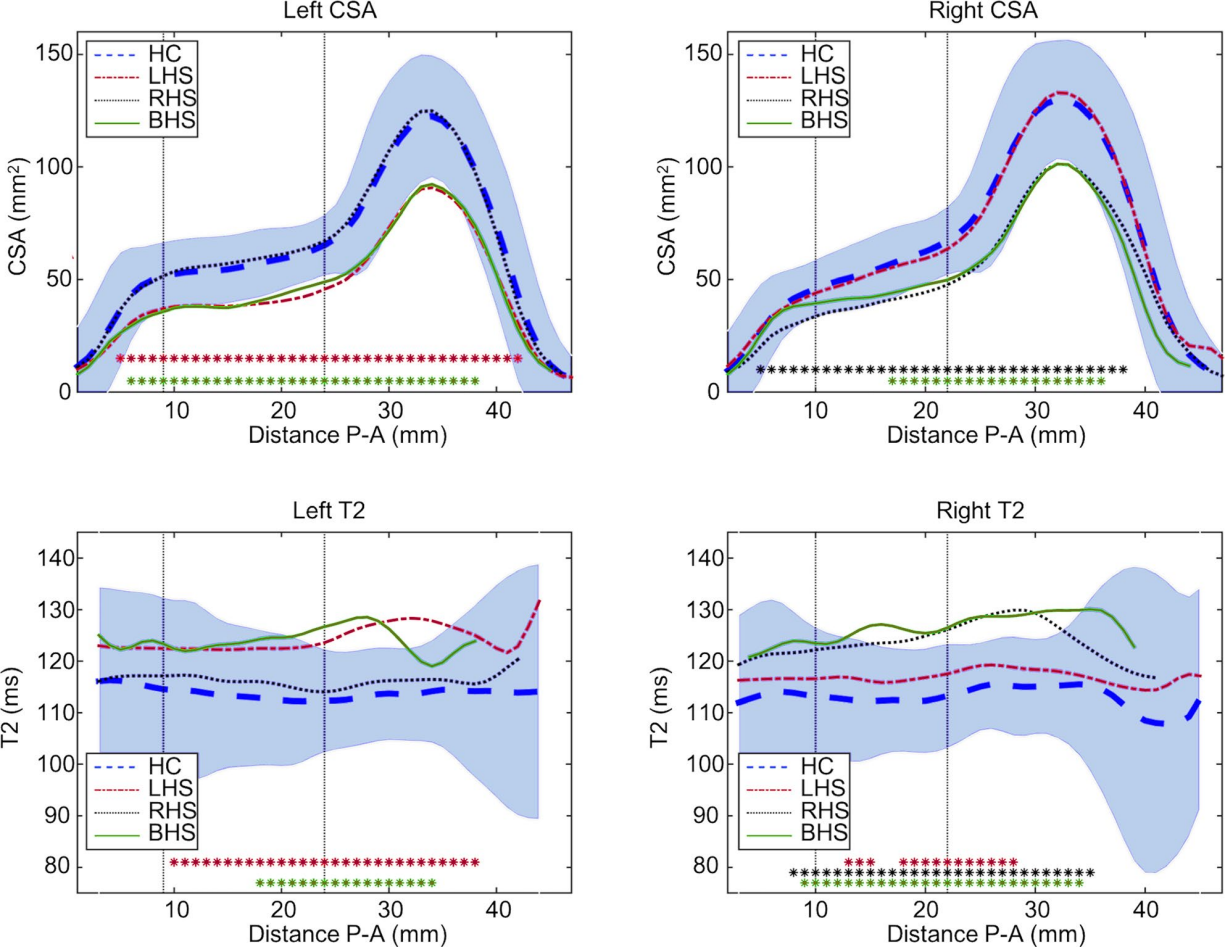
Average cross‐sectional area (CSA; top row) and T2 (bottom row) profiles per subject group. The blue dashed line is the average of the healthy controls (HC), and the blue shaded area the normative range from these controls. The average profiles for each of the three patient groups (left hippocampal sclerosis [LHS] in red, right hippocampal sclerosis [RHS] in black, and bilateral hippocampal sclerosis [BHS] in green) show decreased CSA along much of the length of the hippocampus. The asterisks indicate that at that point the patient profile was below the normative range of the controls (mean ± 1.96 standard deviations). The vertical dotted lines indicate the transitions between head, body, and tail of the hippocampus. P‐A, posterior‐anterior

Average hippocampal volumes were reduced ipsilaterally and qT2 values were increased ipsilaterally in RHS and bilaterally in LHS patients, whereas there were bilateral volume and qT2 changes in BHS patients (Table S2). Comparing the CSA and qT2 profiles of the three patient groups to the normative data shows that on average sclerotic hippocampi had significantly decreased CSA along a large proportion of the length (Figure 3). On a group level, the ipsilateral hippocampus in LHS was more atrophic than the ipsilateral hippocampus in RHS, with 86.4% versus 77.3% of the points along the profile significantly smaller (Figure 3). Similarly, in BHS, the left hippocampus was more atrophic than the right (76.7% vs 48.8%). This asymmetry was less obvious in the ipsilateral qT2 values of unilateral HS. In line with the statistically significant increase in overall contralateral qT2 in LHS (Table S2), 34.1% of points along the profile were significantly elevated. In BHS, a bigger portion of the right hippocampus (76.5%) had increased qT2 than of the left (48.6%), contrasting with the more widespread CSA loss on the left than on the right.

Dividing the hippocampus into a tail, body, and head regions revealed similar findings, with only volumetric changes ipsilaterally in all three regions, and bilateral volumetric reductions everywhere except for the tail of the right hippocampus. For qT2 values per region, there were increases in all three parts ipsilaterally in unilateral HS, and in bilateral body and head in BHS. Contralaterally, LHS had increased qT2 in the body and head, with only the left head showing increased qT2 in RHS. This spatial pattern is matched when investigating how many patients have affected region metrics compared to the normative range from controls (Table S3).

**Figure 4:**
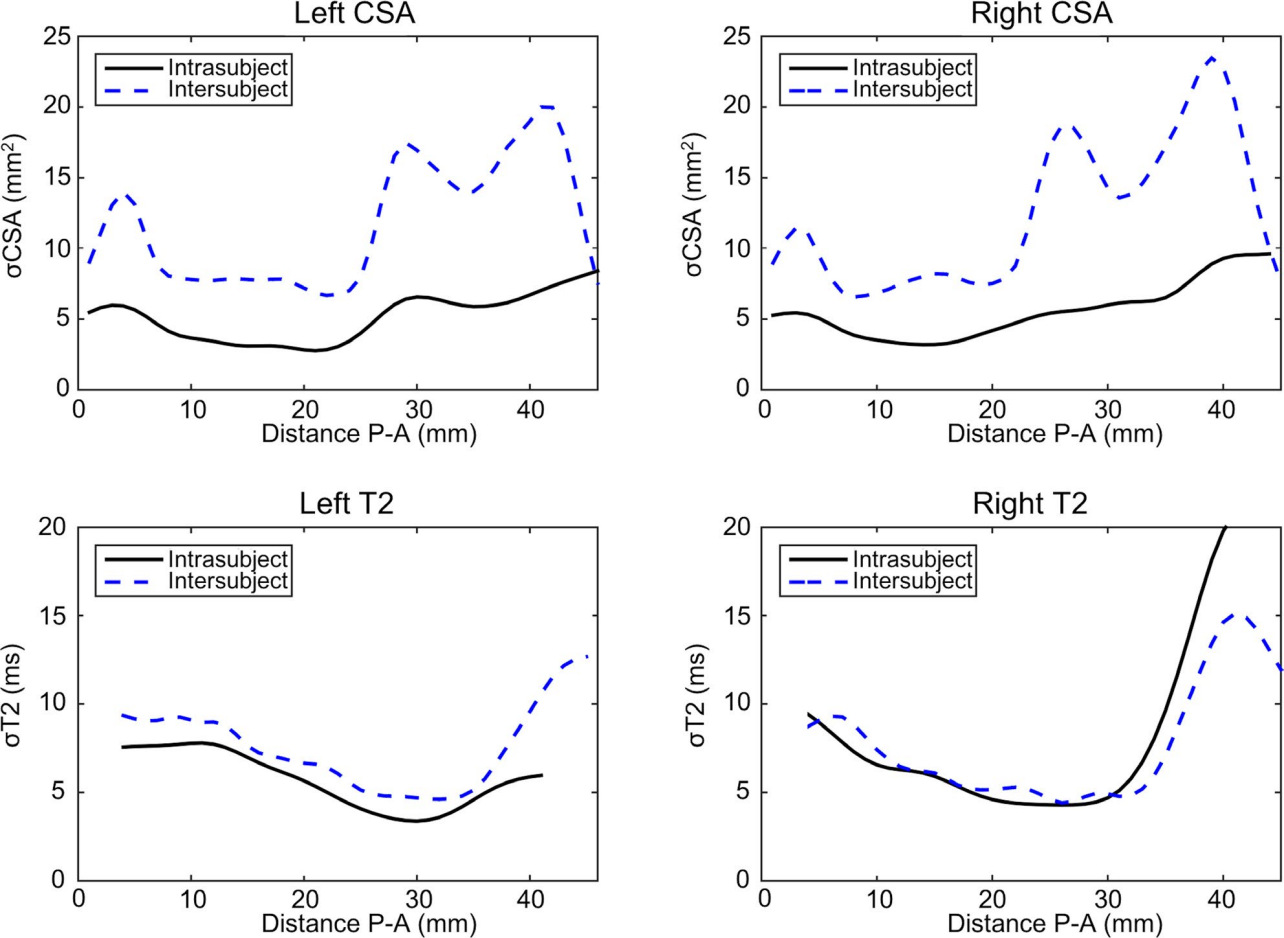
Comparisons of intersubject variability (population‐based standard deviation) in cross‐sectional area (CSA; top row) and T2 (bottom row) compared to intrasubject variability (scan‐rescan) along the length of the hippocampus in 20 controls. P‐A, posterior‐anterior; σ, standard deviation

From the control group, 20 subjects were scanned twice for test‐retest analysis of both the imaging protocols and the analysis methods. A comparison of the standard deviation over the entire control population (n = 111) and the intrasubject scan‐rescan variation show the same spatial patterns, with CSA repeatability much higher than intersubject variation (Figure 4).

**Figure 5:**
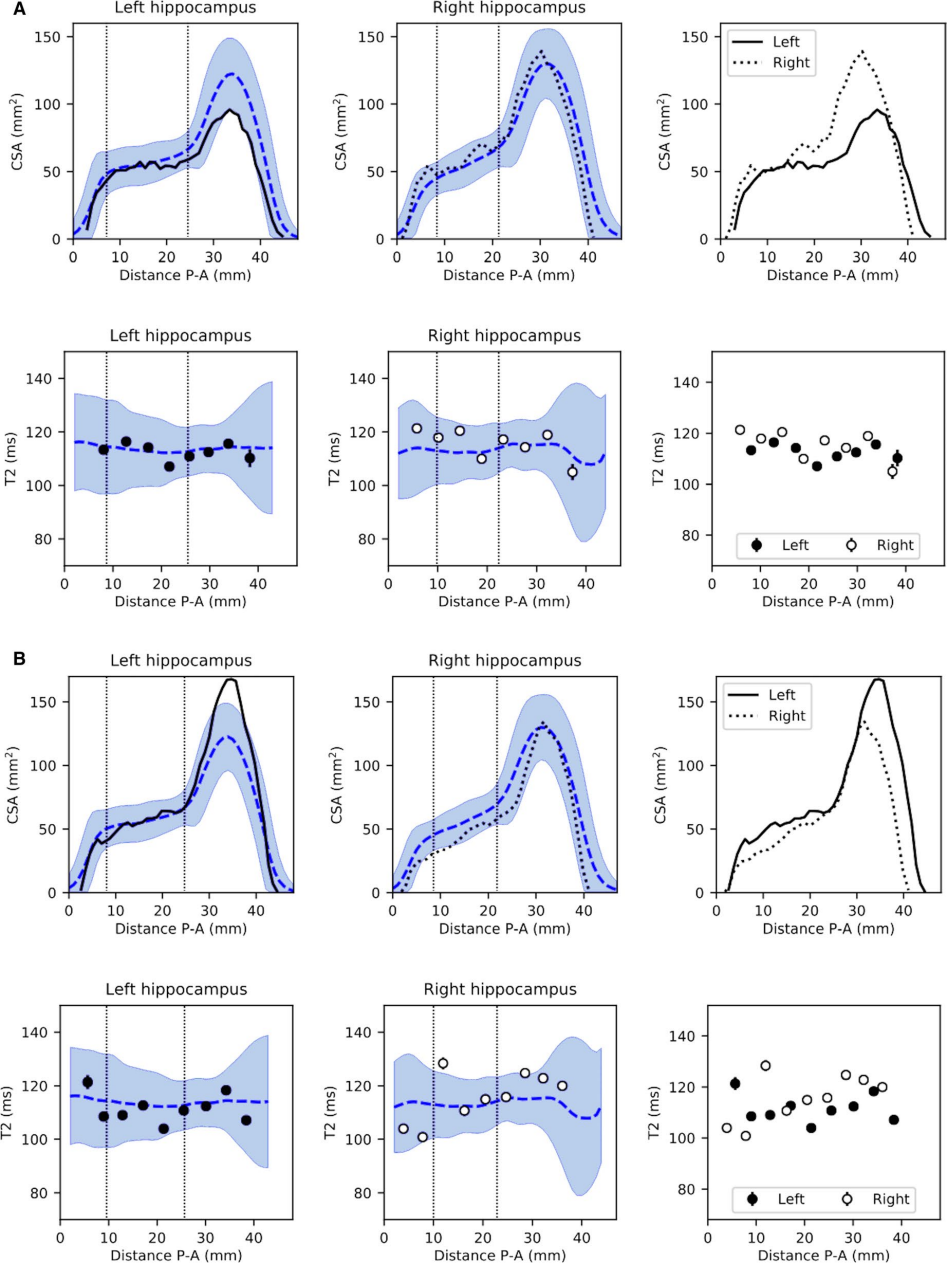
Example profiles of radiologically defined unilateral hippocampal sclerosis (HS) without volume or quantitative T2 (qT2) abnormalities at the whole‐hippocampal level. A, This patient with left HS had a hippocampal volume just within the normative range (2.40/2.84 mL ipsi‐/contralateral, control range = 2.40‐3.39 mL) and normal qT2 (112.8/116.4 milliseconds ipsi‐/contralateral, control range = 108.5‐123.8 milliseconds). Cross‐sectional area (CSA) and T2 profiles show a clear volume asymmetry primarily anteriorly, confirmed with a volume of the head below the normative range for this area (1.35 mL, control range = 1.37‐2.02 mL). B, Patient 2, with right HS, had normal volumes (2.41/3.29 mL ipsi‐/contralateral, control range = 2.40‐3.39 mL) and qT2 (118.9/112.2 milliseconds ipsi‐/contralateral, control range = 108.5‐123.8 milliseconds). CSA and T2 profiles show a clear anterior CSA asymmetry, despite being within the control range. P‐A, posterior‐anterior

Regarding whole‐hippocampal volumes, the ipsilateral hippocampus was significantly atrophic in 29 of 32 (90.6%) of both LHS and RHS cases, with four of five (80%) and five of five (100%) of left and right hippocampi in BHS patients, respectively, atrophic. For whole‐hippocampal qT2, ipsilateral hippocampi had significantly elevated qT2 in 18 of 32 (56.3%) LHS and 21 of 32 (65.6%) RHS patients, with three of five (60%) of left and five of five (100%) of right hippocampi affected in BHS. Figure 5 shows example CSA and T2 profiles for one LHS and one RHS patient in whom no whole‐hippocampus abnormalities were detected. These profiles demonstrate the benefit of analyzing volumetry and qT2 values in more detail, that is, identifying focal abnormalities that would otherwise go undetected.

**Figure 6:**
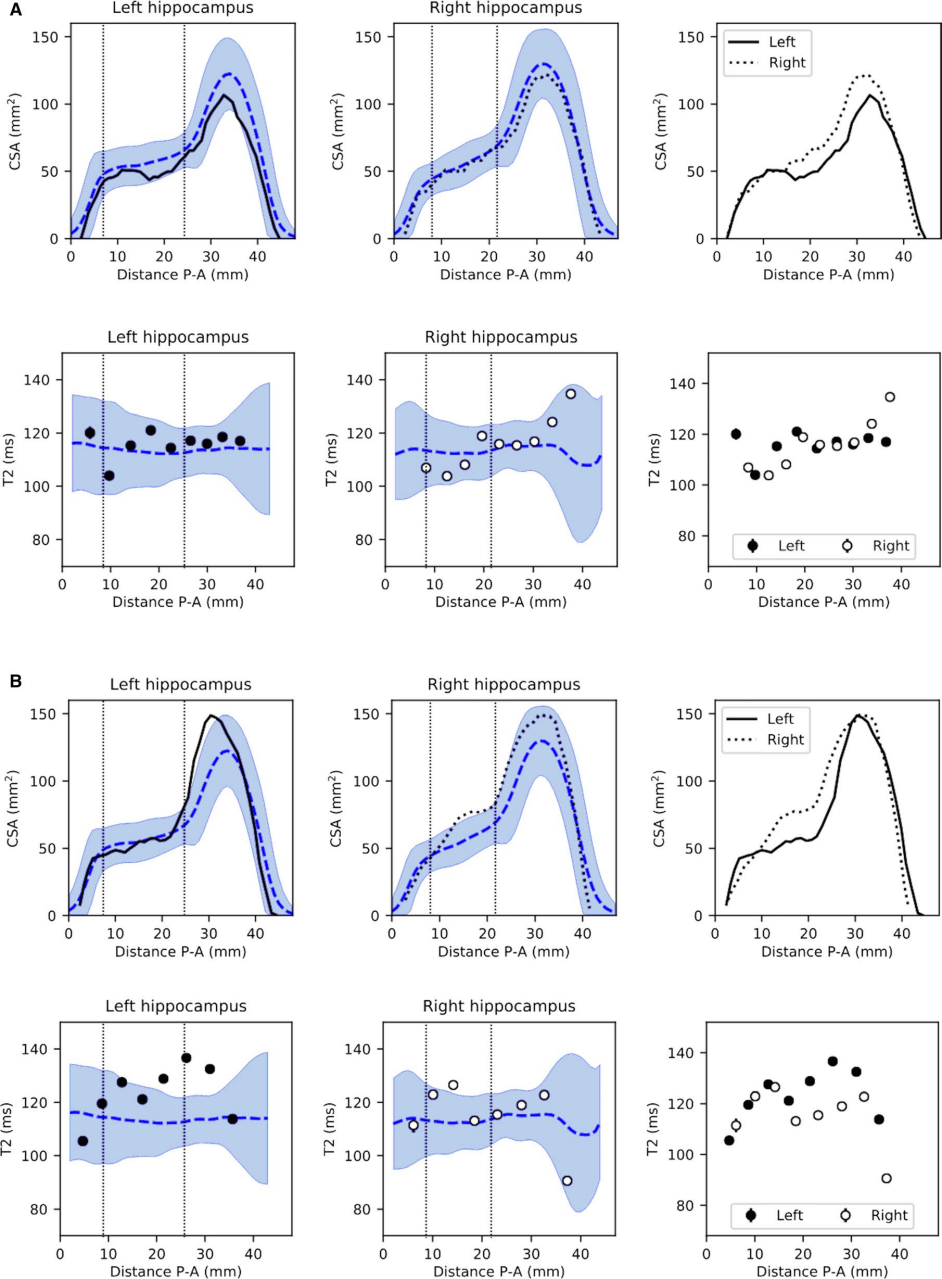
Example cases without radiological diagnosis of hippocampal sclerosis (HS) but with HS on histopathology. A, Patient 2 from Table S1, with left temporal lobe epilepsy (TLE) and normal hippocampal volumes (2.52/2.93 mL for ipsi‐/contralateral, control range = 2.40‐3.39 mL) and quantitative T2 (qT2; 116.3/117.1 milliseconds for ipsi‐/contralateral, control range = 108.5‐123.8 milliseconds). The cross‐sectional area (CSA) profiles show asymmetry along a large proportion of the length of the hippocampus, but with no significant difference in hippocampal head or body volumes. No qT2 abnormalities were observed anywhere along the profile. B, Patient 4 from Table S1 with left TLE and normal hippocampal volumes (2.69/3.01 mL for ipsi‐/contralateral, control range = 2.40‐3.39 mL) and qT2 (123.5/115.1 milliseconds for ipsi‐/contralateral, control range = 108.5‐123.8 milliseconds). CSA profiles show slightly larger than normal volumes along the head and body of the hippocampus, with asymmetry along the body with predominantly lower CSA in the ipsilateral left hippocampus. This corresponds with significantly elevated ipsilateral qT2 values on three consecutive slices, resulting in increased qT2 in the head and body of the hippocampus. P‐A, posterior‐anterior

In the five patients in whom no radiological diagnosis of HS had been made, but resection did reveal histopathological evidence of HS, three cases had dual pathology including HS, and two only HS (Table S1). In two of these patients, the CSA and qT2 profiles were indicative of hippocampal abnormalities (Figure 6). In one patient, midhippocampal atrophy without T2 change was present (Figure 6A). In another patient, signal alterations were remarked upon in radiological review but not deemed clinically significant. Increased T2 was identified with three slices of 4‐mm thickness, with a corresponding localized relative volume reduction (Figure 6B).

## 4 DISCUSSION

In this study, we presented an automated processing framework to evaluate localized hippocampal volumetry and T2 relaxometry abnormalities by visualizing them along the AP axis. We observed group differences between LHS, RHS, and BHS with respect to a large group of healthy controls. These differences in CSA and qT2 are widespread and provide a more detailed representation of the quantitative imaging compared to single values for each hippocampus. We showed that hippocampal profiling can provide additional information compared to whole‐hippocampal measurements, supporting the radiological diagnosis of HS in cases with subtle or focal abnormalities (Figures 5 and 6).

### 4.1 Group differences

There was no evidence for different patterns of atrophy between the affected hippocampi in those with unilateral and BHS, with very similar CSA profiles in the left hippocampus in LHS and BHS, and the right hippocampus in RHS/BHS (Figure 3). We found that in unilateral HS the significantly increased qT2 in the contralateral side (Table S2) is a mild widespread increase, thus providing further support for either drug‐related or seizure‐related changes.^5^, ^20^

### 4.2 Spatial variability

Previous studies of qT2 profiling in the hippocampus found a spatial gradient with qT2 higher anterior than posterior in both controls and patients with HS.^14^ Our results do not show such gradients. The methodology is significantly different between Woermann et al^14^ and this work, with imaging and processing improved over time. In our current work, (1) image resolution is higher (voxel volume = 0.74 mm^3^ vs 4.40 mm^3^), (2) we sample the entire hippocampus rather than a small manually placed region, (3) we exclude any partial volume voxels (as in Winston et al^13^), and (4) we had a bigger control population (111 vs 20), all of which could potentially remove the apparent spatial gradient seen in Woermann et al.^14^

The widespread abnormalities observed in this work (Figure 3) are concordant with evidence from both histopathology and imaging research indicating abnormalities in the CA1‐CA3 subfields and dentate gyrus, which run most of the length of the hippocampus (eg, Briellmann et al,^21^ von Oertzen et al,^22^ Stefanits et al^23^); for comparison of the orientations of these subfields to the CSA profiles, please see Figure S1. Using automated tools for hippocampal subfield segmentation,^24^, ^25^ group‐level differences in these subfields are also observed,^26^ but patient‐specific results at 3 or 7 T have so far remained inconclusive as to the use in either improved detection of HS or prediction of postoperative seizure outcome.^27^, ^28^, ^29^ Even when manual subfield delineation did provide a distinction between HS types 1 and 2, automated subfield volumetry did not.^30^

Increased variability of both CSA and qT2 in the control population around the hippocampal head is also seen in the test‐retest analyses and could be caused by residual imperfections in correcting for different lengths of hippocampi. That the overall scan‐rescan variability is 25%‐30% of the intersubject variation is an indication of how reproducible these CSA profiles are. The intrasubject and intersubject variation in the T2 profiles is almost identical, in both spatial variation along the long axis and magnitude. The higher scan‐rescan variability here is likely to come from different slice positions, with 4‐mm slices inherently causing greater scan‐rescan variability than the isotropic 1‐mm acquisition of the 3D T1. The increased variability in qT2 in the hippocampal head is expected to arise from the underlying anatomy, where the folded structures include small layers of cerebrospinal fluid that increase voxelwise qT2, which our partial volume correction^13^ might not solve fully.

### 4.3 Generalization

This methodology has been made publicly available online, by extending the existing HIPPOSEG Web‐based service (http://niftyweb.cs.ucl.ac.uk/program.php?p=HIPPOPROF) to include CSA profiling.^12^, ^15^ To account for interscanner differences in acquisition protocols, we have added two publicly available MRI datasets to the online tool to almost triple the normative database to enhance generalizability to other centers (see online Appendix S1 for more details). The T2 relaxometry sequence is likely to be more varied with scan setup, and the lack of use outside of dedicated epilepsy imaging centers means there are no available datasets online. We have therefore not included this in our online tool.

### 4.4 Limitations

One of the limitations of the methodology used arises from the inherent issue of modeling the T2 relaxometry as a single qT2 value per voxel. This disregards any partial volume effects and increases the variability in the measurements, especially in the head of the hippocampus. The 4‐mm‐thick slices result in increased partial volume effects, but were necessary to achieve sufficient signal‐to‐noise ratio for reliable qT2 quantification. This study used a dual‐echo T2‐mapping sequence, which has been demonstrated to have precise and reproducible results in hippocampal T2 mapping, with good power to distinguish normal from abnormal hippocampal tissue in the range of T2 values relevant for HS.^13^, ^31^, ^32^ Although the pure accuracy of a dual‐echo sequence is not equal to that of the longer multiecho sequences traditionally used, there is a strong correlation between dual‐echo and 16‐echo T2 values,^31^ making the dual‐echo sequence a robust tool to use in a clinically feasible scan time.

Validation of these tools is complicated by a lack of a ground‐truth of the whole hippocampus. First, hippocampal resections for mesial TLE typically only resect the anterior 2 cm of the hippocampus,^33^ limiting the available histology. We therefore recommend the use of this methodology as an adjunct to expert radiological and neurological review.

The patient selection criteria for the large cohort used in this study were based on review of radiological reporting, selecting confirmed or suspected HS cases. This may also have resulted in demonstrating the profiling method more as a tool for improved characterization rather than improved diagnosis of subtle HS, even if improved sensitivity over whole‐hippocampal quantification was demonstrated (Figure 5).

### 4.5 Clinical utility

The potential clinical utility of this method is threefold. First, it identifies subtle, especially focal anterior, HS that may be overlooked by visual reading. Second, it offers the possibility of a restricted hippocampal resection, sparing structurally normal hippocampal tissue that may be contributing usefully to memory function.^34^ Third, it identifies subtle BHS that may not be reported on visual reading and that may militate against hippocampal resection.

### 4.6 Implications and future work

The presented automated subject‐specific analysis has been designed to integrate into a quantitative imaging setup of 3D T1 and T2 relaxometry already recommended for routine clinical imaging in TLE patients.^10^, ^35^ The personalized approach taken in this work is specifically intended to facilitate integration into patient‐based clinical research settings, hence the visualization approaches taken to compare profiles to a normative database. In this, it is different from many recent approaches designed for group‐based analyses.^26^, ^36^, ^37^, ^38^, ^39^ Other single‐subject approaches to evaluate HS do exist, with the most similar in setup using normalized FLAIR intensity to detect HS, which has good outcome but is limited in our original approach in only having a single "global" value per hippocampus.^40^, ^41^ We suggest that our proposed method may increase sensitivity, specificity, and/or localization (as indicated in Figures 5 and 6) when used in radiological reporting or epilepsy surgery multidisciplinary team discussions. Further evaluation in other patient populations is now warranted.

## 5 CONCLUSIONS

Localized volumetry and T2‐relaxometry measures of the hippocampus are possible to extract in a reliable and reproducible manner. CSA profiling is freely available online for widespread use.

## CONFLICT OF INTEREST

None of the authors has any conflict of interest to disclose. We confirm that we have read the Journal's position on issues involved in ethical publication and affirm that this report is consistent with those guidelines.
