# Supplementary material for: Hippocampal profiling: Localized magnetic resonance imaging volumetry and T2 relaxometry for hippocampal sclerosis
Source: Epilepsia. 2019 Dec 24;61(2):297–309. doi: 10.1111/epi.16416 (PMC7065164; doi:10.1111/epi.16416)
Supplement: Supplementary file 1 [file EPI-61-297-s001.pdf]

Supplementary material

Hippocampal subfield segmentation

The healthy control shown in Fig. 2 was processed with FreeSurfer v6.0.0 using the automated hippocampal segmentation method (Iglesias et al., 2015) and aligned with the template image to visualise the association of the subfields with the hippocampal CSA profiles (Fig. S1).

Generalisation

For generalisability to an online tool, we included two publicly available 3T imaging datasets: Alzheimer's Disease Neuroimaging Initiative (ADNI) and Track-HD (Tabrizi et al., 2012). TRACK-HD T1-weighted image volumes were acquired using a 3D MPRAGE acquisition sequence on 3.0 T Siemens and Phillips scanners with a voxel size of 1.10×1.10×1.10 (Philips) and 1.07×1.07×1.10 (Siemens). This included a total of 98 subjects (age μ±σ 46.6±10.7, range 23.0-65.7 years; 43M/55F). The 3T ADNI data were acquired at multiple scanners and scans were only excluded for image quality reasons. Voxel size for these 3D MPRAGE/IR-FSPGR acquisition varied between 1.00×1.00×1.20 to 1.05×1.05×1.20 mm (see http://adni.loni.usc.edu/methods/documents/mri-protocols/ for imaging details), leading to a total of 217 subjects. To match sample sizes to the Chalfont and Track-HD populations, 109 randomly selected subjects were taken (age μ±σ 73.7±5.7, range 59.9-86.0 years; 50M/59F).

The methodology of processing these additional datasets was identical to the main manuscript. Fig. S2 shows the comparison of the normative ranges for both left and right hippocampi in individual plots. Fig. S3 shows the averages and standard deviations of the three populations in a single plot. Comparing the three datasets, the mean CSA along the hippocampal long axis varies very little despite the generally higher age in the ADNI database (Fig. S3a,b). Similarly, the same regions of increased variability are seen (Fig. S3c,d) and little difference in the magnitude of variation. As a result, we feel justified in combining all three of these populations.

***Supplementary Table S1**: Details of patients without HS as radiological diagnosis*

	Age at scan	Age at surgery	Radiological diagnosis	Pathological report
Patient 1 (F)	27.0 y	27.4 y	R amyg lesion – DNT?	DNT (GG,GR 1), HS (EFS)
Patient 2 (F)	28.1 y	28.8 y	L amyg lesion – DNT?	DNT (GNT), HS type 1
Patient 3 (F)	48.2 y	48.4 y	Subtle increase in T2 signal in R amyg and hippocampal head	HS type 2
Patient 4 (F)	37.6 y	37.7 y	L hippocampal body asymmetry, no convincing signal alteration	HS type 1
Patient 5 (M)	60.8 y	62.3 y	Cavernoma abutting left temporal horn	Cavernoma, mild HS type 3

HS = hippocampal sclerosis ; F = female, m = male; R = right; L = left; Amyg = amygdala; DNT = Dysembryoplastic neuroepithelial tumour;

Supplementary Table S2*: Volume and qT2 of whole hippocampi*

	HC (n=111)	LHS (n=32)	RHS (n=32)	BHS (n=5)
Left volume (ml)	2.87 ± 0.26	1.92 ± 0.35*	2.83 ± 0.21^	1.95 ± 0.23*†
Right volume (ml)	2.92 ± 0.25	2.82 ± 0.29	2.07 ± 0.28*^	2.14 ± 0.31*^
Left qT2 (ms)	112.7 ± 11.4	125.1 ± 6.9*	115.9 ± 4.7^	124.6 ± 7.3*†
Right qT2 (ms)	113.4 ± 11.5	117.8 ± 3.1*	126.0 ± 6.2*^	127.7 ± 3.5*^

HC = healthy controls; LHS = left HS; RHS = right HS; BHS = bilateral HS; qT2 = quantitative T2; * indicates p<0.001 with respect to HC; ^ p<0.001 with respect to LHS; † p<0.001 with respect to RHS.

Supplementary Table S3*: Percentage of patients with volume and qT2 abnormality per region*

	LHS (n=32) head / body / tail	RHS (n=32) head / body / tail	BHS (n=5) head / body / tail
Left volume	87.5 / 87.5 / 25	0.0 / 6.3 / 0.0	80 / 80 / 40
Right volume	6.3 / 6.3 / 9.4	78.1 / 71.8 / 43.8	100 / 40 / 20
Left qT2	84.4 / 43.8 / 0.0	18.8 / 15.6 / 0.0	80 / 60 / 0
Right qT2	6.3 / 18.8 / 0.0	71.9 / 68.8 / 0.0	100 / 80 / 0

LHS = left HS; RHS = right HS; BHS = bilateral HS; qT2 = quantitative T2

Supplementary figures

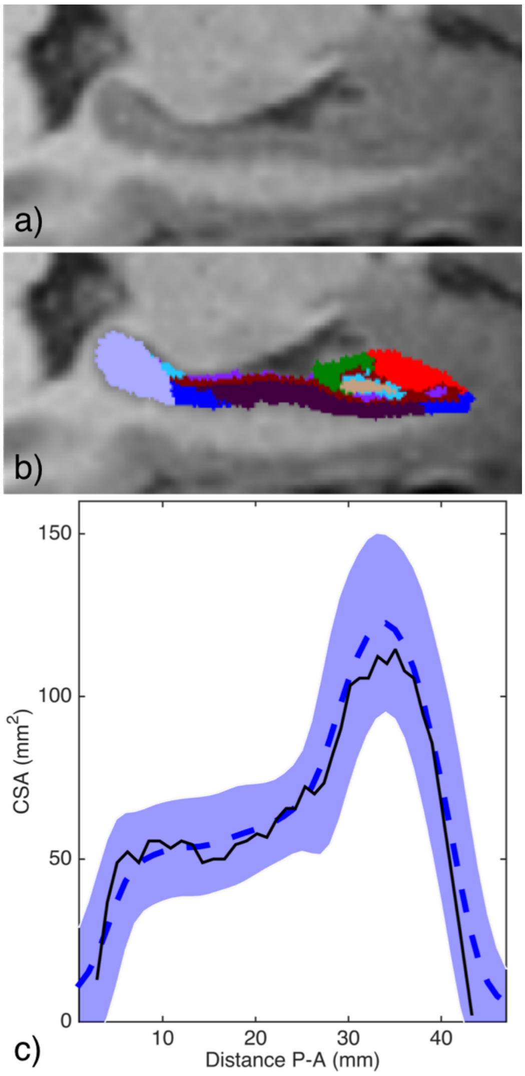

Figure S1: A sagittal slice through the left hippocampus (a), with the subfields colour-coded (b), and the associated cross-sectional area (CSA) profile (c). The subfield colours are: CA1=red, CA2=, CA3=green, CA4=brown, molecular layer=maroon, dentate gyrus=aqua, presubiculum=dark purple, subiculum=dark blue, tail=light purple.

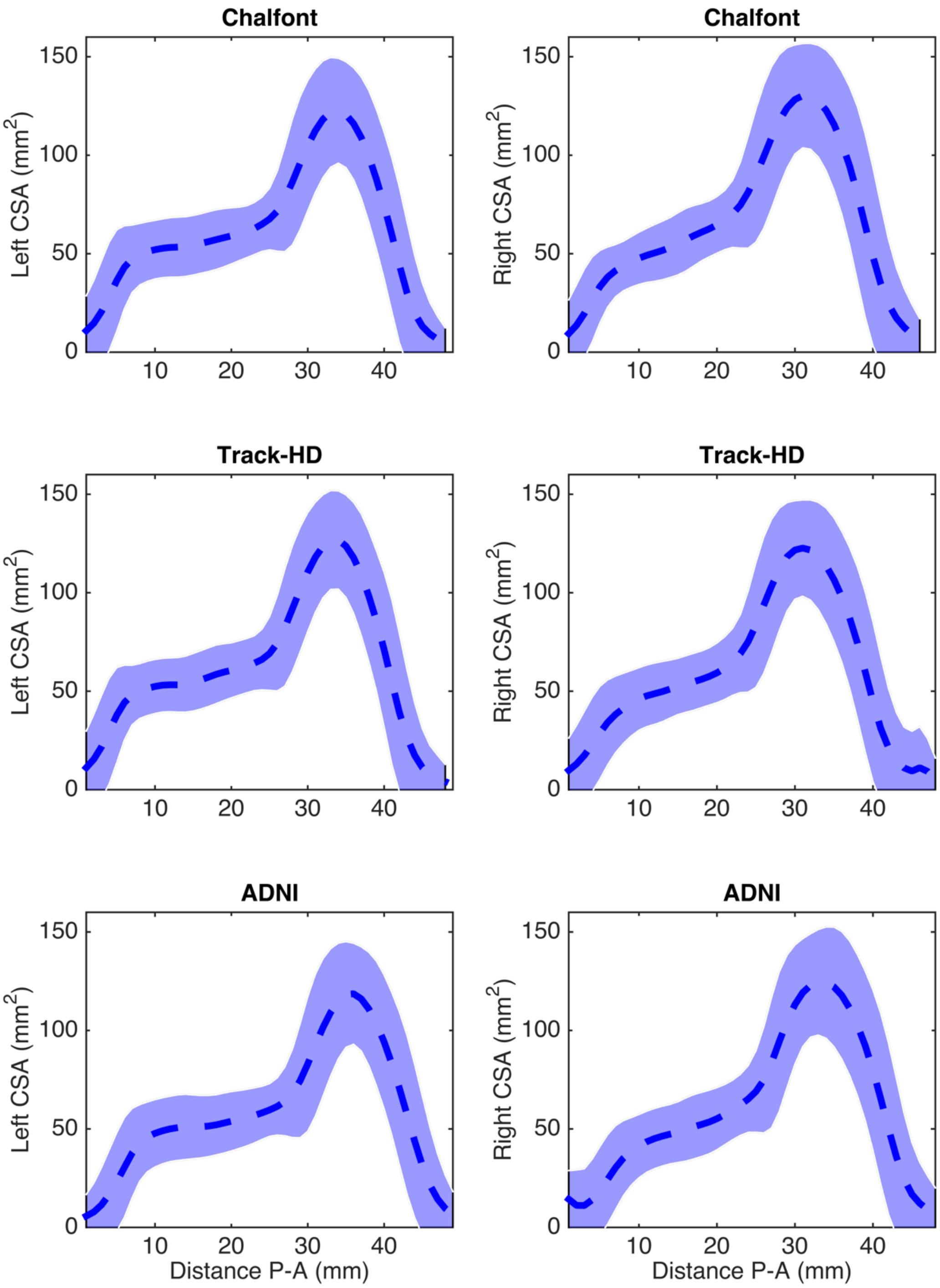

Figure S2: Mean and normative ranges shown for cross-sectional area (CSA) plot from three different healthy control cohorts: a local Chalfont one, from the Track-HD study, and from ADNI.

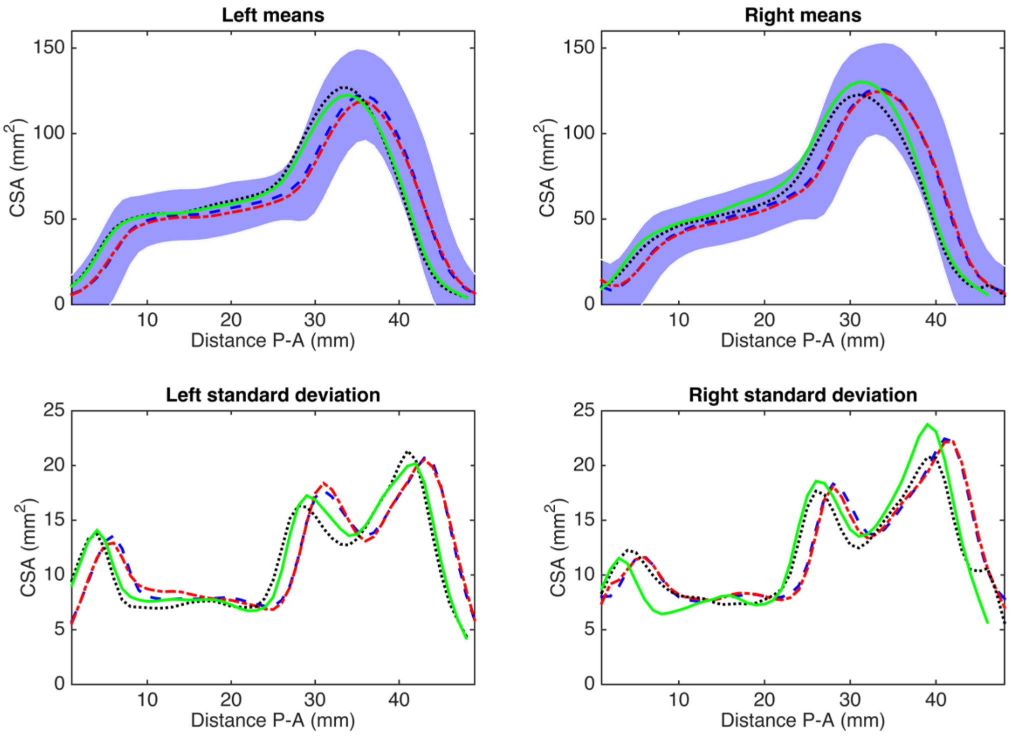

Figure S3: The normative range (blue shaded area) of the entire healthy control population from the three different cohorts, with the mean shown as a dashed blue line, for the CSA of the left (a) and right (b) hippocampus. The mean CSA profiles for the three cohorts are shown: local cohort (solid green line), ADNI (red dashed-dotted line), and Track-HD (black dotted line). Panels c and d show the standard deviations along the PA-axis, with the same colour-encoding per line.
